# Complete chloroplast genome of *Holboellia grandiflora* Réaubourg (Lardizabalaceae), a plant with an edible fruit

**DOI:** 10.1080/23802359.2022.2087542

**Published:** 2022-06-24

**Authors:** Yan Li, Qinxiang Chang, Caijuan Zhang, Huimin Li, Pengguo Xia

**Affiliations:** a Department of Art Design, Taiyuan University, Taiyuan, PR China; b Key Laboratory of Plant Secondary Metabolism and Regulation of Zhejiang Province, College of Life Sciences and Medicine, Zhejiang Sci-Tech University, Hangzhou, PR China

**Keywords:** chloroplast genome, *Holboellia grandiflora* Réaubourg, phylogenetic analyses, Lardizabalaceae

## Abstract

*Holboellia grandiflora* Réaubourg (Lardizabalaceae) is an evergreen twining perennial woody vine. To our knowledge, this is the first report on the complete chloroplast genome sequence of *H. grandiflora*. The complete chloroplast genome sequence was 157,811 bp in length and contained a large single-copy region of 86,554 bp and a small single-copy region of 18,975 bp. A pair of inverted repeats of 26,141 bp were included. It contained 130 genes, comprising 37 transfer *RNA* genes, and eight ribosomal *RNA* genes, as well as 85 coding sequences (CDSs). The GC content of the complete chloroplast genome sequence was 38.7%. The phylogenetic tree showed a close relationship among the three species of *Holboellia* (H. *grandiflora, H. angustifolia*, and *H. latifolia*). These findings provide a reference for phylogenetic relationships and assessment of the genetic structure of the Lardizabalaceae family.

*Holboellia grandiflora*, Réaubourg 1906, which belongs Lardizabalaceae, is an evergreen twining perennial woody vine mainly distributed at an altitude of 800–3000 m (Flora of China Editorial Committee 2001). *Holboellia grandiflora* does not currently exist on the IUCN Red List. Its pistillate and staminate flowers are purple and the staminate ones are green, respectively. The fruit of H. *grandiflora* can be consumed and also used for wine making. In addition, its seeds can be pressed into oil. Currently, this species is widely distributed in the wild and has not been developed and used. It is well adapted to environmental conditions and has strong resistance to stress (Tao et al. 2013).

Fresh leaf tissue of *H. grandiflora* was sampled from a mountain located in Longwang Town, Ningshan County, Ankang City, Shaanxi Province (33°10′39.88″N,108°31′28.14″E, and altitude 853 m). The voucher specimen was preserved at the Herbarium of Xi’an Botanical Garden (voucher number: *Xun Lulu* et al. *00780*, Lulu Xun, xunlulu20032006@126.com).

Total genomic DNA was extracted from fresh leaves using a modified CTAB method (Doyle 1987). The sequencing library was constructed using the Illumina Hiseq6000 platform with an insert size of approximately 490 bp. In total, raw PE reads of approximately 1.86 Gb were generated and approximately 1.85 Gb of clean PE reads were obtained after using fastp application (Chen et al. 2018) to trim and filter them. The complete chloroplast genome of *H. grandiflora.* was assembled using the software NOVOPlasty version 2.7.2 software (Dierckxsens et al. 2017) (Brussels, Belgium). The chloroplast genome was determined using Geneious Prime software, referring to the sequence of *H. angustifolia* (NC053741), and was submitted to GenBank (GenBank ID MW970138). The complete chloroplast genome sequence of *H. grandiflora* was 157,811 bp in length, with a large single-copy region of 86,554 bp and a small single-copy region of 18,975 bp. A pair of inverted repeats of 26,141 bp were included. It contained 130 genes, comprising 37 *tRNA* genes, 8 *rRNA* genes, and 85 coding sequences (CDSs).

Based on the complete chloroplast genomes of *H. grandiflora* and related species, we constructed a phylogenetic tree using maximum likelihood (ML) phylogenetic analyses (Figure 1); phylogenetic tree was constructed using IQTREE version 1.6.7 (Nguyen et al. 2015) (Vienna, Austria) based on 13 complete chloroplast genome sequences of Lardizabalaceae and *Euptelea pleiosperma* (NC029429) as the outgroup, under K3Pu + F+G4 model with 1000 bootstrap replicates. Phylogenetic analysis revealed that *H. grandiflora* has a strong sister relationship with *H. angustifolia* (NC053741) and *H. latifolia* (MH394375).

**Figure 1.**
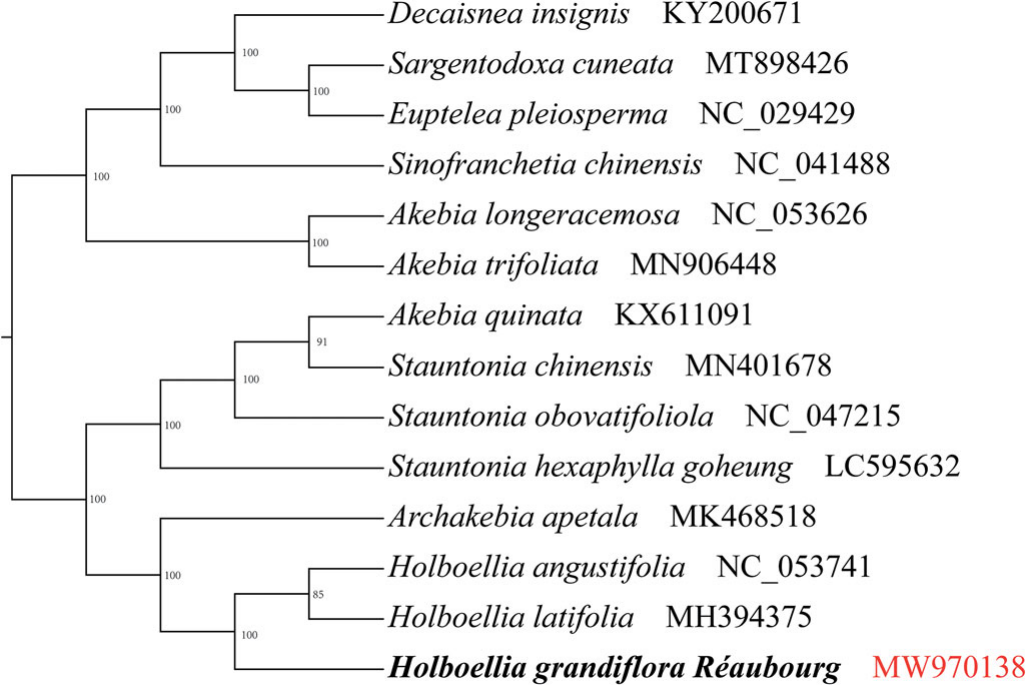
Phylogenetic tree showing the relationship between *Holboellia grandifolia* and 12 Lardizabalaceae species with *Euptelea pleiosperma* (NC029429) as an outgroup. Phylogenetic tree was constructed based on the complete chloroplast genomes using maximum likelihood (ML) with 1000 bootstrap replicates. Numbers in each the node indicated the bootstrap support values.

The gene content, GC content, and gene order of *H. grandiflora* were found to be similar to those of *H*. *angustifolia* (Wu et al. 2019). Additionally, a total of 49 microsatellites (SSRs) were identified in the *H. grandiflora* cp genome using MISA. Among them, 37 were located in the LSC regions, whereas 2 and 8 were found in the IR and SSC regions, respectively. The majority of the SSRs (44/49) were A or T repeats, which was consistent with the A/T-richness in the complete cp genome (Xuan et al. 2013). The complete chloroplast genome sequence provides the necessary data for the study of organ development genes and phylogenetic studies of the Lardizabalaceae family. The findings of this study could be useful for further studies on *H. grandiflora* and its various applications.

### Ethical approval

Research and collection of plant material were conducted according to the guidelines provided by Xi’an Botanical Garden. Permission was granted by Hangzhou Academy of Agricultural Sciences to carry out research on the species.

## Author contributions

Y.L. and P.X. conceived and designed this study. C. Z. and H. L. conducted analysis. P.X. and C.Z. contributed the analytical methods. Y.L. wrote the manuscript. P.X. edited the manuscript. All authors have read and agreed to the published version of the manuscript.

## Data Availability

The data that support the findings of this study are openly available in NCBI (https://www.ncbi.nlm.nih.gov) GenBank with the accession number MW970138. The associated BioProject, SRA, and BioSample funding numbers are PRJNA723130, SRR14278902, and SAMN18805688, respectively.
